# Posterior Reversible Encephalopathy Syndrome in a Pediatric Burn Patient: A Case Report

**DOI:** 10.7759/cureus.90792

**Published:** 2025-08-23

**Authors:** Shishir Shouri, Jignesh Sharma, Aditya VT, AMBER KUMAR, Girish Bhatt

**Affiliations:** 1 Pediatrics, All India Institute of Medical Sciences, Bhopal, Bhopal, IND; 2 Pediatric Medicine, All India Institute of Medical Sciences, Bhopal, Bhopal, IND; 3 Pediatrics and Child Health, All India Institute of Medical Sciences, Bhopal, Bhopal, IND

**Keywords:** central line-associated bloodstream infections (clabsi), convulsion, hypertension\, pediatric burn care, posterior reversible encephalopathy syndrome (pres)

## Abstract

Posterior reversible encephalopathy syndrome (PRES) is a rare clinico-radiological condition characterized by convulsions, headache, visual disturbances, and altered consciousness. PRES is often associated with immunosuppressive treatment, autoimmune disorders, hypertension, and renal illness, but it is rarely reported in pediatric burns. We report a four-year-old boy who presented with a history of hot water burns that affected 20% of his total body surface area (TBSA). He subsequently experienced right-sided hemifocal seizures, right hemiparesis, and a persistent fever three months after injury. MRI Brain revealed bilateral parieto-occipital hyperintensities and microhemorrhages consistent with PRES. Central line-associated bloodstream infection (CLABSI) with *Candida tropicalis* and hypertension were identified as the major contributing factors. Antiepileptics, antihypertensives, antifungals, nutritional rehabilitation, and skin grafting were all part of the management. Symptoms resolved within two weeks, and the patient was discharged without neurological deficits. This case underscores the importance of maintaining a heightened level of clinical suspicion for PRES in pediatric burn patients presenting with neurological symptoms. Reversing neurological impairments and achieving positive results depend on early detection, neuroimaging, and timely treatment of underlying triggers, which include infections and hypertension. The paper contributes to the limited literature on pediatric burn-related PRES, emphasizing the importance of multidisciplinary care and clinical monitoring in resource-limited settings.

## Introduction

Posterior reversible encephalopathy syndrome (PRES) is a clinico-radiological disorder characterized by reversible white matter edema, primarily affecting the parieto-occipital regions, and manifests with seizures, headache, visual disturbances, and altered consciousness [1]. PRES may occur without overt encephalopathy or severe hypertension; however, common triggers include uncontrolled blood pressure, renal insufficiency, immunosuppressive therapy, and autoimmune disorders [2]. While reports of PRES in adults are frequent, it is rare in children, particularly in the context of burn injuries [3]. Pediatric burn patients face unique challenges, including sepsis and prolonged inflammation, which may predispose them to PRES through disrupted cerebral autoregulation [4]. This case report describes a rare case of PRES in a four-year-old boy with burns, highlighting its pathogenesis, diagnosis, and management to contribute to the limited literature on this topic.

## Case presentation

A four-year-old, developmentally normal, second-born male child out of a non-consanguineous marriage who presented with complaints of fever for three months and right-sided hemifocal seizures along with right-sided hemiparesis for the last three days. He had sustained hot water burns three months back, affecting 20% of the total body surface area (TBSA). He received treatment at an outside facility with intravenous antibiotics, symptomatic management, and two units of packed RBC (PRBC). The presence of persistent fever, along with recent right-sided hemifocal seizures, prompted referral to our facility.

On admission, the patient was febrile (38.5°C), with severe acute malnutrition (weight-for-age z-score <-3 SD) and right-sided hemiparesis. Blood pressure was elevated (130/86 millimetres of mercury (mmHg), >95th percentile + 12 mmHg for age). Seizures were controlled with IV levetiracetam (60 mg/kg/day) and IV sodium valproate (60 mg/kg/day). Laboratory findings revealed anemia (hemoglobin 9.5 g/dL), thrombocytopenia (platelet count 26×10³/µL), and elevated CRP (41.95 mg/dL) (Table 1).

**Table 1:** Laboratory trends during hospitalization

Parameter	Day 1	Day 7	Day 14	Day 20	Reference values
WBC (×10³/µL)	8.33	8.86	9.78	7.71	4.0-11.0
CRP (mg/dL)	41.95	97.75	116.03	4.64	<5.0
Hemoglobin (g/dL)	9.5	7.7	7.7	8.9	11.5-15.5
Platelet (×10³/µL)	26	42	69	176	1.5-4.5
Creatinine (mg/dL)	0.36	0.34	0.32	0.29	0.3-1.0
Total bilirubin (mg/dL)	1.37	1.01	0.9	0.7	0.3-1.2
Albumin (g/dL)	3.2	3.11	2.7	2.8	3.5-5.2
Magnesium (mg/dL)	2.23	2.15	2.20	2.26	1.9-2.5
Sodium (mmol/L)	138.43	141.21	139.10	139.35	136-145

Blood and central line tip cultures grew *Candida tropicalis*, which was sensitive to echinocandins, confirming the diagnosis of central line-associated bloodstream infection (CLABSI). Urine culture showed budding yeast cells, prompting escalation to caspofungin. CSF analysis was normal (cell count: three lymphocytes and two polymorphs, protein: 20.6 mg/dL, glucose: 52.27 mg/dL), ruling out meningitis.

**Figure 1:**
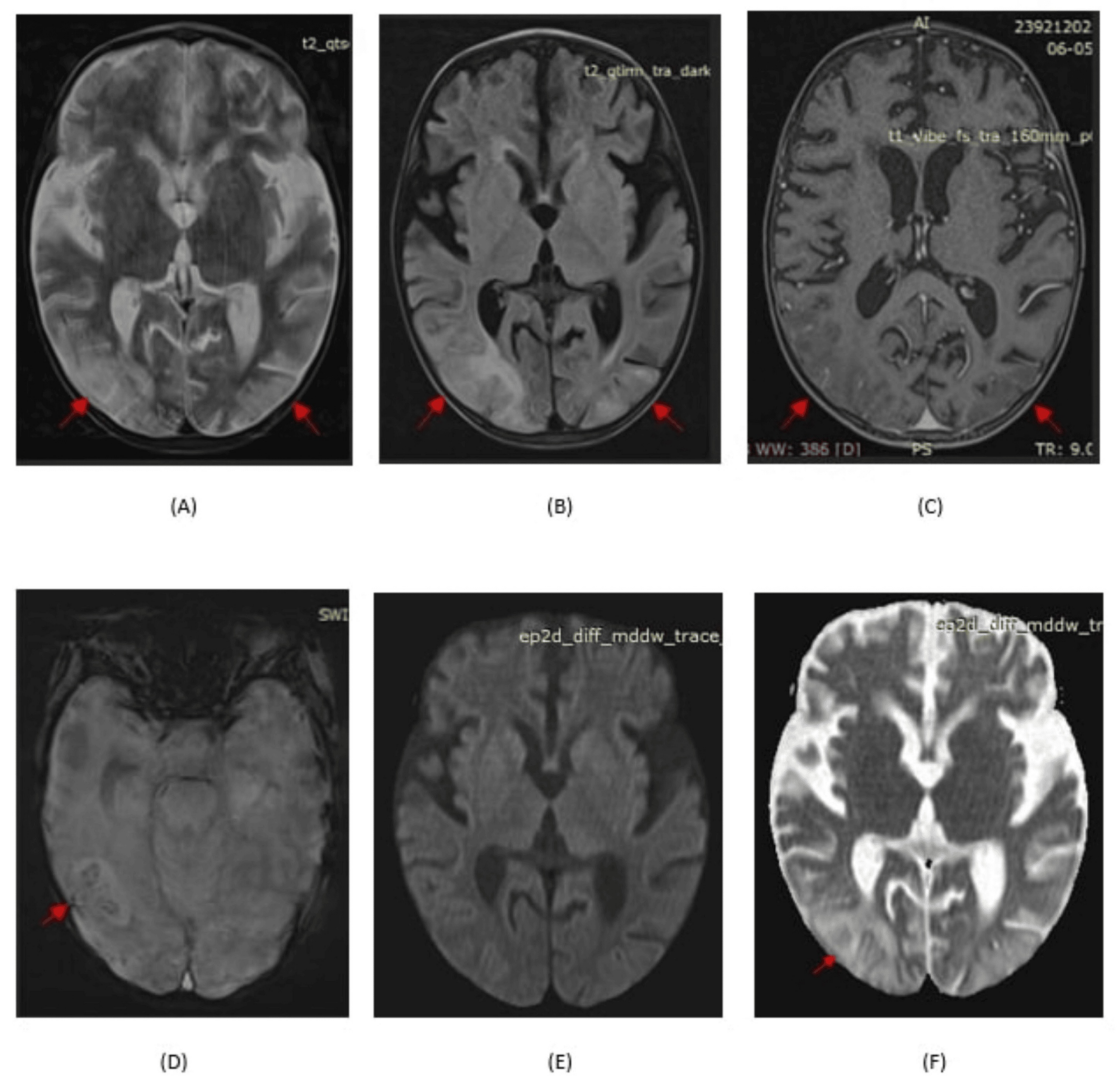
MRI brain showing bilateral parieto-occipital hyperintensities on T2 and FLAIR (A, B), hypointensities on T1 (C), microhemorrhages on SWI (D), no diffusion restriction on DWI (E), and facilitated diffusion on ADC (F), findings consistent with vasogenic edema ADC: apparent diffusion coefficient; FLAIR: fluid-attenuated inversion recovery; DWI: diffusion-weighted imaging; SWI: susceptibility-weighted imaging

**Figure 2:**
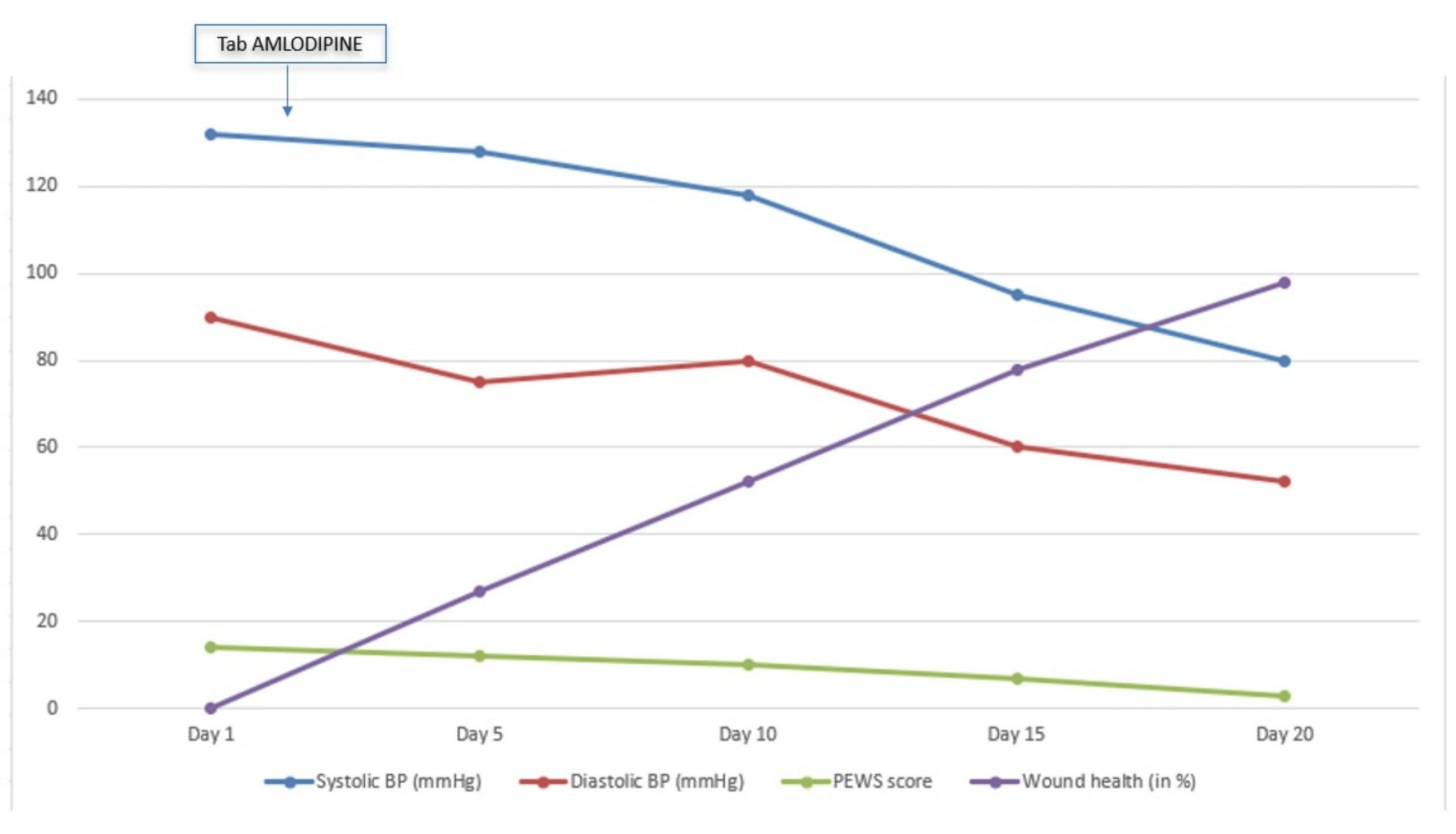
Sequential trends showing normalization of systolic and diastolic blood pressure after amlodipine, progressive improvement in wound healing, and a parallel decline in pediatric early warning score (PEWS) score, indicating overall clinical improvement

**Figure 3:**
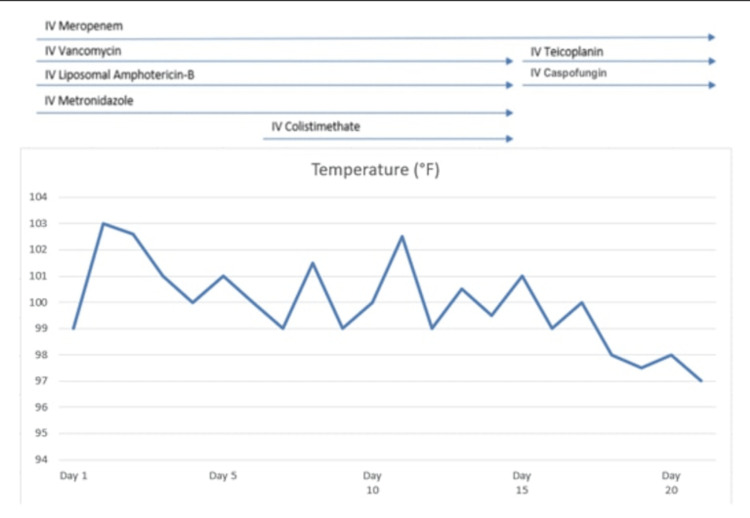
Fever chart showing resolution with caspofungin

Brain MRI showed bilateral parieto-occipital hyperintensities on T2-weighted and fluid-attenuated inversion recovery (FLAIR) sequences, corresponding hypointensities on T1-weighted images, and microhemorrhages on susceptibility-weighted imaging (SWI). Diffusion-weighted imaging (DWI) showed mild hyperintensity without corresponding restricted diffusion on apparent diffusion coefficient (ADC) maps, consistent with vasogenic edema and confirming the diagnosis of PRES (Figure 1). Hypertension was managed with oral amlodipine (0.1 mg/kg/day), tapered as blood pressure normalized (Figure 2). This hemodynamic stabilization coincided with progressive wound healing and a parallel decline in the pediatric early warning score (PEWS), reflecting overall improvement in the patient’s clinical status [5]. Fever subsided following initiation of antifungal therapy, which was continued for 28 days (Figure 3). Wound care included debridement, daily dressings, and skin grafting on day 15. Nutritional rehabilitation involved a high-protein diet. By day 20, seizures resolved, and significant improvements in hemiparesis and laboratory parameters were found (Table 1). Due to financial constraints, follow-up imaging was not performed, but clinical improvement (regular neurological exam) guided discharge.

## Discussion

PRES, first described by Hinchey et al. in 1996, is characterized by reversible vasogenic edema primarily in the posterior regions of the brain, resulting in neurological manifestations [1]. Hypertension in pediatric burn patients is multifactorial, commonly arising from pain and stress-mediated neuroendocrine activation, inflammatory sepsis, and excessive fluid resuscitation, which leads to hypervolemia and increased peripheral resistance [6]. Pediatric PRES is less frequently reported than in adults, with a 2022 systematic review of 449 cases reporting renal disease (36.7%), hematological disorders (18.7%), and autoimmune conditions (14.3%) as the primary cause [2] (Table 2).

**Table 2:** Causes of PRES in pediatrics PRES: posterior reversible encephalopathy syndrome

S. No.	Cause Category	Estimated prevalence (%)	Proposed mechanism	Clinical implications
1	Renal disorders	36.7	Hypertension, uremia, volume overload	Monitor BP and renal function, early dialysis
2	Hematological disorders	18.7	Endothelial injury, hypoxia, treatment toxicity	Manage underlying disease and transfusions
3	Autoimmune diseases	14.3	Vasculitis, immune activation, drug effects	Monitor during flares and immunotherapy
4	Oncologic conditions	10.5	Chemotherapy toxicity, cytokine release	Neuro-monitoring during treatment
5	Hypertensive encephalopathy	5.0	BP exceeds autoregulation capacity	Tight BP control, antihypertensive therapy
6	Infections/sepsis	4.5	Inflammation, endothelial damage	Supportive care, early imaging
7	Medications/toxins	3.2	Drug-induced vasoconstriction or toxicity	Dose adjustment, agent substitution
8	Postoperative/ICU	2.5	BP fluctuations, inflammation	ICU neuro checks, fluid control
9	Burns	2.0	Fluid overload, sepsis, stress	Judicious fluids, early recognition
10	Metabolic disorders	2.0	Electrolyte imbalance affects vasculature	Correct imbalances promptly

Burn-related PRES is exceptionally uncommon, with only two prior reports: a 13-year-old boy with severe burns linked to surgical complications and hypertension, and a 20-year-old female with 41% TBSA burns presenting two months after burn injury [3,4]. These cases suggest burns may trigger PRES through systemic inflammation or vascular dysregulation [7,8].

This four-year-old boy’s case, with 20% TBSA burns, is notable for its complex etiology involving hypertension and sepsis. Hypertension likely induced hyperperfusion, disrupting cerebral autoregulation and causing vasogenic edema [9]. Simultaneous prolonged fever, along with CLABSI, suggests a hypoperfusion mechanism, where sepsis-induced vasoconstriction and hypoxia increase vascular endothelial growth factor (VEGF) expression, leading to increased vascular permeability and ultimately causing vasogenic edema [10,11]. Classical MRI findings of bilateral parieto-occipital hyperintensities on T2-weighted and FLAIR sequences, along with microhemorrhages on SWI, supported the diagnosis of PRES. DWI showed mild hyperintensity without restricted diffusion on apparent ADC maps, consistent with vasogenic edema. Normal magnetic resonance angiography (MRA) findings helped exclude large-vessel vasculopathies, including reversible cerebral vasoconstriction syndrome (RCVS). Furthermore, a normal CSF analysis, as well as improvement in neurological symptoms, distinguished PRES from infectious or inflammatory conditions such as meningitis or encephalitis [12]. Treatment with amlodipine, caspofungin, and antiepileptics (levetiracetam, sodium valproate) resolved hypertension, sepsis, and seizures within two weeks, with nutritional support and skin grafting aiding recovery. Financial constraints limited follow-up imaging, but serial improvement in neurological exams confirmed resolution.

This case highlights the need for a high index of suspicion of PRES in burn patients with abnormal neurological findings, as it may mimic other neurological conditions (e.g., stroke or infections). Early MRI of the brain is the cornerstone of diagnosis, along with multidisciplinary care involving a physician, neurologist, and plastic surgeon. In resource-limited settings, clinical monitoring (e.g., neurological exams, blood pressure tracking) compensates for imaging constraints. This article adds to the limited body of research on burn-related PRES in pediatric patients by promoting early detection and thorough treatment, ultimately improving neurological outcomes.

## Conclusions

This case of PRES in a four-year-old boy with 20% TBSA burns, triggered by hypertension and *C. tropicalis *CLABSI, highlights a rare complication in pediatric burn care. Rapid diagnosis via MRI brain and targeted treatment (e.g., antihypertensives, antifungals, antiepileptics, and wound management) resulted in early resolution of central nervous system (CNS) symptoms. This report contributes to the sparse literature on burn-related PRES in children, with particular emphasis on its reversibility with timely intervention. Key lessons learned include the following. (1) PRES in pediatric burns is unusual and may mimic other neurological disorders such as stroke or CNS sepsis; hence, early MRI brain should be prioritized. (2) The presence of hypertension in the background of CNS symptoms in pediatric burn may be the earliest indicator of underlying PRES.
